# Interventions to prevent antipsychotic-induced weight gain and metabolic complications in individuals with a first-episode psychosis and minimal antipsychotic exposure: a systematic review and meta-analysis

**DOI:** 10.1017/S0033291726104139

**Published:** 2026-04-10

**Authors:** Brian William O’Mahony, Tara Burke, Ruby Hamill, Louisa Gannon, Benjamin I. Perry, Dan Siskind, Tomasz Pawełczyk, Donal O’Shea, Brian O’Donoghue

**Affiliations:** 1 University College Dublin, Dublin, Ireland; 2 University of Birmingham, UK; 3 The University of Queensland, Australia; 4 Medical University of Lodz: Uniwersytet Medyczny w Lodzi, Poland

**Keywords:** antipsychotic, BMI, first episode, metabolic, physical health, psychosis, schizophrenia, side effect, weight gain, prevention

## Abstract

**Background:**

Rapid weight gain commonly occurs following the onset of first-episode psychosis (FEP), leading to cardiometabolic disease. Most weight gain in FEP occurs in the first 3 months of treatment, offering a critical window for prevention. Despite this, most studies aiming to prevent antipsychotic-induced weight gain include people with chronic illness or people who have had lengthy exposure to antipsychotic medication. We aimed to synthesize and analyze the literature on interventions aimed at preventing antipsychotic-induced weight gain.

**Methods:**

We conducted a systematic review in PsycInfo, MEDLINE, CINAHL, and EMBASE of studies that examined the effectiveness of interventions in preventing antipsychotic-induced weight gain in FEP. We examined their effect on weight gain and a range of cardiometabolic markers.

**Results:**

We screened 2,092 articles, 13 of which were eligible. Behavioral interventions, all three of which consisted of a multidisciplinary team approach, resulted in a mean of 3.05 kg less weight gain than treatment-as-usual (95% CI 1.36 kg to 4.73 kg). Pharmacological interventions displayed marked clinical and statistical heterogeneity, with each of the seven trials in adults using a different pharmacological intervention. Few studies collected comprehensive data on metabolic health. Only two pharmacological studies, and five studies in total, have been published since 2010.

**Conclusions:**

Despite the importance of preventing weight gain in FEP, there have been few recent studies investigating this topic. Our results indicate that multidisciplinary team interventions are effective in preventing weight gain in FEP and should be offered to all patients.

## Introduction

Higher mortality in people with severe mental illness is well recognized, with an estimated 15-year reduction in life expectancy, of which cardiometabolic conditions are a key component (Laursen, 2011; Plana-Ripoll et al., 2019). This mortality gap is widening, pointing to a failure of interventions to prevent or treat cardiovascular risk factors (Nielsen, Uggerby, Jensen, & McGrath, 2013). Globally, people with severe mental illness have increased rates of overweight and obesity (Afzal et al., 2021), which are major mediators of cardiovascular disease incidence and mortality (Correll et al., 2017; Peeters et al., 2003).

Weight gain is common in people following the onset of psychotic illnesses, and is influenced by genetic predisposition, diet, lifestyle, and the use of antipsychotic medication (Correll, Lencz, & Malhotra, 2011; Dipasquale et al., 2013; Pillinger et al., 2017; Vancampfort et al., 2017). A key finding of the literature on antipsychotic-induced weight gain (AIWG) is that most of the total weight gain occurs in the first 3 months of exposure to the medication and is particularly pronounced in people who are drug-naïve (Alvarez-Jimenez et al., 2008; Pérez-Iglesias et al., 2014; Zipursky et al., 2005). Therefore, any intervention aimed at preventing AIWG should be delivered at the time of initiation of the first antipsychotic medication or with minimal prior exposure to such treatments. Systematic reviews to date on the prevention of AIWG, while informative, are limited by the inclusion of studies involving individuals who were not all experiencing a first episode or who had previous exposure to antipsychotic medications (Agarwal, Stogios, Faulkner, & Hahn, 2023; Nyboe, Lemcke, Møller, & Stubbs, 2019; Yu et al., 2024).

The weight gain and adverse metabolic effects caused by antipsychotics are a result of their widespread binding to monoaminergic receptors in both the central nervous system and peripheral tissues. For example, blockade of histamine-1 (H_1_) and Serotonin 2c (5HT_2c_) receptors has been linked to increases in appetite, while second-generation antipsychotics’ effect on receptors in the pancreas, liver, and skeletal muscle leads to dysregulation of glucose and lipid homeostasis (Carli et al., 2021). Antipsychotics’ propensity to increase appetite compounds the unhealthy diet often seen in people with psychotic disorders (Dipasquale et al., 2013), and their sedative effects are likely contributors to the sedentary behavior of people with psychotic disorders (Stubbs, Williams, Gaughran, & Craig, 2016).

Early intervention in psychosis services was founded with the recognition that this critical period offers an opportunity to minimize the adverse effects of psychosis on an individual experiencing first-episode psychosis (FEP). The same principle of early intervention should be applied to physical health, given that metabolic changes are far more difficult to reverse than to prevent (Lemieux & Després, 2020).

Given this, we aimed to comprehensively review the literature on, and analyze the effectiveness of, interventions in preventing weight gain in individuals experiencing a first episode of psychosis who are antipsychotic-naïve or have had minimal exposure. We also aimed to assess the effectiveness of interventions to prevent other metabolic complications, specifically the components of metabolic syndrome, including hypertension, hyperglycemia, and dyslipidemia.

## Methods

### Types of studies

We included randomized controlled trials (RCTs), non-randomized controlled trials, and other interventional study designs such as pre–post studies. We excluded non-interventional observational studies, cohort studies, case studies, and case series.

### Types of participants

We included studies whose participants were experiencing a FEP, and who had been exposed to antipsychotics for less than 4 weeks. If a study allowed a longer exposure to antipsychotic medications as part of their inclusion criteria, we contacted the authors and asked if they could provide data pertaining to participants with less than 4 weeks of exposure. We set no restrictions on participant demographic characteristics or duration of untreated psychosis.

### Search strategy

We searched the following databases: PsycInfo, MEDLINE, CINAHL, and EMBASE. We constructed the search using three core concepts: (1) the population (schizophrenia and psychosis), (2) the outcomes of interest (metabolic health factors), and (3) a specifier for the illness stage (early or first episode). For each concept, a combination of controlled vocabulary terms (e.g. MeSH, Emtree, CINAHL Subject Headings) and free-text keywords was used. Where appropriate, we ‘exploded’ controlled vocabulary terms to capture all related sub-headings. Supplementary Appendix A details the full search strategy. We inspected references of all included studies for further relevant studies. The latest original search date was 20th December 2024. We then carried out another full search on 16th October 2025, prior to submission, at which point we identified no new relevant studies.

### Study selection

Two independent reviewers screened citations and full texts and resolved disagreements by consensus or by discussing with a third reviewer. When adequate information was not available, we attempted to contact the authors of the study concerned for clarification.

### Data extraction

Data were first extracted on 14th January 2025. We chose mean weight gain in kilograms (kg) as our outcome measure for the primary objective, prevention of weight gain, and used mean difference in the meta-analysis. We also collected data on ‘clinically significant’ weight gain (≥7% weight increase from baseline); body mass index (BMI), waist circumference, and markers of metabolic health, for example, lipids, fasting glucose, and HbA1C. Where a study utilized a different unit of measurement, we converted to standardized metric units, for example, kilograms and mmol/l.

When not provided in a paper, we estimated the mean difference between groups. When within-group standard deviations of mean differences were not provided, we estimated this standard deviation using confidence intervals, p-values, and/or results of Student’s t-test. Where necessary, we imputed the standard deviation of the mean difference by using the correlation coefficient from similar studies within the review (Chi, Li, Chen, & Kang, 2023). We weighed studies in the meta-analysis using inverse variance and conducted the meta-analysis using the RevMan web version (Review Manager, 2024).

We used the *Cochrane Handbook for Systematic Reviews of Interventions* to assess the risk of bias in study quality (Cumpston et al., 2019). Where possible, we presented data on last observation carried forward (LOCF), and presented completer (per-protocol) analyses only when intention-to-treat data were not available.

We examined clinical heterogeneity by comparing study populations and intervention types. We examined methodological heterogeneity by reviewing study design features. If we detected substantial clinical or methodological variability, we synthesized results narratively. We used the I^2^ statistic to investigate heterogeneity between studies (Higgins, Thompson, Deeks, & Altman, 2003). We considered an I^2^ statistic estimate of >75% to be indicative of substantial heterogeneity (Deeks, Higgins, Altman, & Group, 2019). For our meta-analysis, we used a fixed-effects model. We conducted sensitivity analyses where relevant, which included investigating different follow-up intervals and comparing analyses using a random-effects model.

## Results

**Figure 1.**
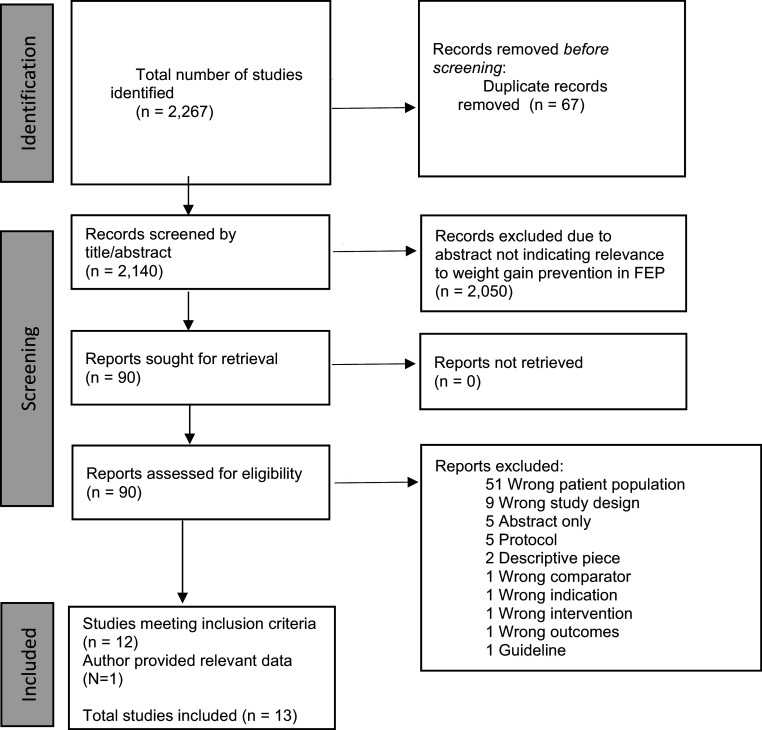
PRISMA flowchart of study identification.

Search results are shown in Figure 1. Our search identified 2,207 articles. After removing 67 duplicates, 2,140 unique articles remained for abstract screening. We then reviewed the full texts of 90 studies. Of these, 10 studies strictly met our inclusion criteria (Arman, Sadramely, Nadi, & Koleini, 2008; Curtis et al., 2016; Kang et al., 2024; Narula, Rehan, Unni, & Gupta, 2010; O’Donoghue et al., 2022; Poyurovsky et al., 2002, 2004, 2007, 2013; Wu et al., 2008).

Alvarez-Jimenez et al. had an inclusion threshold of less than 6 weeks of antipsychotic exposure, but indicated in their paper that no patients had more than 4 weeks of exposure to antipsychotic medication (Alvarez-Jimenez et al., 2006). This paper and its follow-up paper thus met our inclusion criteria (Álvarez-Jiménez et al., 2010). We identified 23 papers whose inclusion criteria indicated that they may have collected data on patients who met our own criteria. We contacted each corresponding and lead author of these papers and received data from Pawełczyk, Grancow-Grabka, Żurner, and Pawełczyk (2021). We were thus able to add these two studies (one of which was split into two papers) to our original 10 studies, allowing us to include 13 papers from 12 studies in our review.

### Study characteristics

Table 1 presents the general characteristics of each study. We included 11 randomized control trials (Alvarez-Jimenez et al., 2006; Arman et al., 2008; Kang et al., 2024; Narula et al., 2010; O’Donoghue et al., 2022; Pawełczyk et al., 2021; Poyurovsky et al., 2002, 2004, 2007, 2013; Wu et al., 2008). The earliest study was published in 2002, and the most recent in 2024. Studies varied in sample size (14–77) and location. Eight studies focused on pharmacological methods to prevent AIWG, three studies focused on behavioral methods, and one study used continuous theta-burst stimulation (cTBS). Only two of the studies of pharmacological interventions were published after 2010.

**Table 1.** General characteristics of identified studies

Author Year Country	Population	% Male	Mean Age(SD)	Follow-up length	No. of patients (analyzed)	Intervention	Control
Alvarez-Jimenez et al. (2006, 2010), Spain	Outpatients, FEP, <6 weeks APS exposure^a^	75.4%	28.8 (12.1)	2 years (1 year in meta-analysis	61 (61)	Early Behavioral Intervention, 10–14 individual sessions with a clinical psychologist, Goals: Engagement & assessment, education, dietary counselling, exercise program, behavior therapy	Weekly sessions: General advice on weight management without structured intervention.
Arman et al. (2008), Saudia Arabia	Inpatients, <20 years old, FEP, drug naïve, prescribed risperidone	65.6%	10.1 (3.6)	12 weeks	49 (32) Per protocol	Risperidone 2–6 mg OD, Metformin 500 mg BD, For 12 weeks	Risperidone 2–6 mg OD For 12 weeks
Curtis et al. (2016), Australia	Outpatients, FEP, <4 weeks of APS exposure Medically fit enough to exercise	60.7%	20.7 (2.2)	12 weeks	49 (28) Per protocol	Health coaching by nurse specialist, dietician support, exercise program tailored by exercise physiologist, peer wellness coaches. enhanced pharmacovigilance	Individual case management with medical assessment and antipsychotic prescription based on clinical guidelines
Kang et al. (2024), China	Inpatients, FEP, drug-naïve, no other mental disorders, comorbid severe physiological diseases, or pregnant/lactating women;	13.9%	25.8 (7.4)	5 days	39 (36) ITT	25 sessions (5 days) continuous theta-burst stimulation 50 Hz bursts of three pulses every 200 ms. Intensity of 80% of the individual resting motor threshold	25 sessions over 5 days, with the coil tilted 90° to the scalp to avoid stimulation of the motor cortex
Narula et al. (2010), India	FEP, drug naïve, no other neuropsychiatric illness, not taking SSRI, or mood stabilizer	65.7%	31.1 (9.8)	12 weeks	72 (67) Per protocol	Olanzapine 5–20 mg OD, Topiramate 50–100 mg OD For 12 weeks	Olanzapine 5–20 mg OD For 12 weeks
O’Donoghue et al. (2022), Australia	Outpatients, 16–25 years old, FEP, < 30 days of APS exposure	50.6%	19.4 (3.4)	12 weeks	77 (61)	Education from a physical health nurse about diet, physical activity, & metabolic side-effects of medication. Referred to an exercise physiologist and dietician	As with the intervention group, but the referrals were handled directly by the case manager or psychiatrist
Pawełczyk et al. (2021), Poland	Inpatients, FEP, no intellectual disability or previous head injury	58.1%	23.7 (4.9)	26 weeks	71 (43 had <4 weeks of APS exposure) ITT	2.2 g of polyunsaturated fatty acids: (1.32 g/day of eicosapentaenoic acid & 0.88 g/day of docosapentaenoic acid	Prescribed antipsychotic and placebo containing olive oil
Poyurovsky et al. (2002), Israel	Inpatients, FEP, <4 weeks of APS exposure, no medical illness affecting body weight	78.3%	25.3 (6.5)	8 weeks	30 (24) Per protocol	Fluoxetine 20 mg OD & Olanzapine 10 mg nocte	Olanzapine 10 mg nocte & placebo
Poyurovsky et al. (2004), Israel	FEP inpatient prescribed olanzapine. <4 weeks of APS exposure, no mood or substance use disorder, no medical illness affecting body weight	57.1%	23.6 (1.7)	6 weeks	14 (14)	Famotidine 40 mg OD & olanzapine 10 mg nocte	Olanzapine 10 mg nocte & placebo
Poyurovsky et al. (2007), Israel	FEP inpatients prescribed olanzapine. <4 weeks of APS exposure, BMI of <30, no mood disorder or suicidal behavior, no medical illness affecting body weight	64.4%	29.9 (7.9)	6 weeks	59 (41) Per protocol with ITT sensitivity analysis	Reboxetine 4 mg BD & Olanzapine 10 mg per day	Olanzapine 10 mg per day & placebo
Poyurovsky et al. (2013), Israel	FEP inpatient prescribed olanzapine. <4 weeks of APS exposure, BMI <30, no mood disorder or suicidal behavior, no medical illness affecting body weight	81.4%	32.5 (9.2)	6 weeks	54 (43) ITT	Reboxetine 4 mg BD & Betahistine 48 mg TID & Olanzapine 10 mg per day	Olanzapine 10 mg nocte & placebo
Wu et al. (2008), China	Inpatients, FEP, no previous APS exposure, no illicit drugs in last 3 months, no diabetes, dyslipidemia, or cardiovascular disease	54.1%	25.1 (3.7)	12 weeks	40 (37) ITT	Metformin 250 mg TID & Olanzapine 15 mg nocte Standardized diet: 7980–9250 kilojoule/day	Olanzapine 15 mg nocte & placebo TID Standardized diet: 7980–9250 kilojoule/day

*Note:* FEP, first-episode psychosis, APS, antipsychotic, ITT, intention-to-treat.

a The paper specified that, although their inclusion criteria was <6 weeks of APS exposure, no participant had more than 4 weeks of APS exposure.

One study was conducted in children (n = 32) (Arman et al., 2008), with a mean age of 10.1 years. All other studies were conducted in adults aged between 18 and 65 years. Each of the eight pharmacological intervention studies (total n = 316) and the cTBS study (n = 39) was assessed in an inpatient setting, and participants in each of the three behavioral intervention studies (n = 187) were assessed in an outpatient setting.

Pharmacological interventions were clinically heterogeneous. Among adult participants, no two studies assessed the same intervention. While the behavioral interventions were broadly characterized by a multidisciplinary approach, they differed in their theoretical frameworks and delivery personnel. Álvarez-Jiménez et al. (2006) utilized an Early Behavioral Intervention based on cognitive-behavioral principles. The intervention was delivered by clinical psychologists over 8–14 sessions, with the aim of modifying cognitive triggers and enhancing self-control over energy intake. In contrast, Curtis et al. (2016) implemented the Keeping the Body in Mind (KBIM) program, which was a high-intensity, holistic life-skills intervention delivered by a team of nurses, dietitians, and exercise physiologists. A unique feature of KBIM was the inclusion of youth peer wellness coaches who acted as role models during supervised gym sessions and practical life-skill activities, such as shopping tours and cooking classes. Finally, O’Donoghue et al. (2022) evaluated the Physical Health Assistance in Early Psychosis (PHAstER) study, which integrated a dedicated physical health nurse into existing teams to act as a central care coordinator. This model focused on motivational interviewing and facilitated referrals to existing allied health services.

The type of antipsychotic used differed among studies. Among pharmacological interventions, six included only patients who were prescribed olanzapine (Narula et al., 2010; Poyurovsky et al., 2002, 2004 2007, 2013; Wu et al., 2008), as did Kang et al’s study of cTBS (Kang et al., 2024). Arman et al.’s (2008) study of metformin in adolescents included only patients who were prescribed risperidone. Each of the three behavioral interventions included patients who were prescribed a variety of antipsychotics (Alvarez-Jimenez et al., 2006; Curtis et al., 2016; O’Donoghue et al., 2022), as did Pawełczyck et al.’s (2021) study of polyunsaturated fatty acids.

### Control groups

Table 1 describes the control intervention for each study. Among studies including only participants who were prescribed olanzapine, similar weight gain was noted among those with a 6-week follow-up (4.77–5 kg) Poyurovsky et al., 2002, 2004, 2007, 2013), and among those with a 12-week follow-up (6.03 kg and 6.87 kg) (Narula et al., 2010; Wu et al., 2008). The control group in Kang et al’s study gained 1.1 kg in just 5 days (Kang et al., 2024). Mean weight gain was lower in studies that used a variety of antipsychotics. The control group of inpatients in Pawełczyk et al.’s (2021) study gained 2 kg at 13 weeks and 4 kg at 26 weeks. All participants in the three behavioral studies were outpatients with FEP with less than 4 weeks of antipsychotic exposure. Despite these similarities, there was a significant difference in weight gain between the three control groups at 3-month follow-up (F = 6.615, p < 0.01). The control group in O’Donoghue et al.’s (2022) study gained less weight (2.9 kg) than the control group in Curtis et al.’s (2016) study (7.8 kg) or Alvarez-Jimenez et al’s (2006) study (7 kg).

### Risk of bias and study quality

Table 2 shows our assessment of each study’s risk of bias across domains. Arman et al.’s (2008) paper included only patients with a diagnosis of first-episode schizophrenia, but with a mean age of 10.1 (SD 3.6). We did not identify any conflicts of interest that may have impacted study quality.

**Table 2.** Risk of bias

Author	Random sequence generation	Allocation concealment	Blinding of personnel and participants	Blinding of outcome assessment (weight)	Blinding of outcome assessment (others)	Incomplete outcome data	Selective reporting	Other bias
Alvarez-Jimenez et al. (2006, 2010)	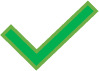	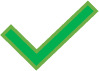	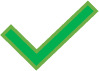	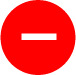	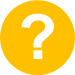	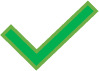	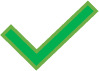	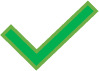
Arman et al. (2008)	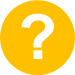	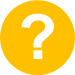	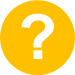	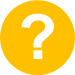	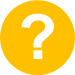	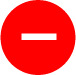	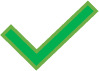	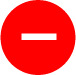
Curtis et al. (2016)	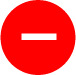	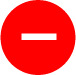	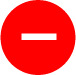	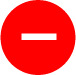	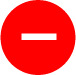	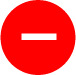	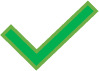	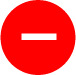
Kang et al. (2024)	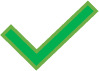	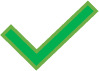	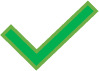	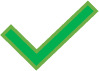	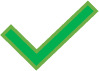	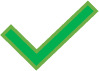	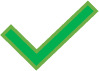	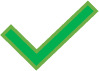
Narula et al. (2010)	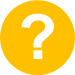	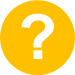	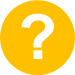	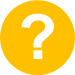	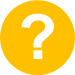	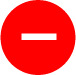	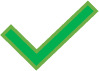	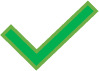
O’Donoghue et al. (2022)	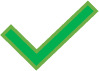	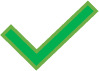	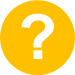	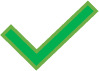	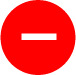	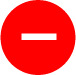	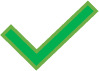	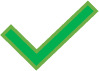
Pawełczyk et al. (2021)	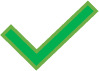	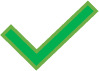	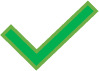	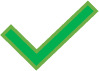	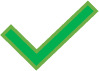	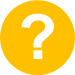	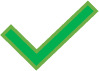	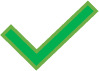
Poyurovsky et al. (2002)	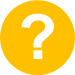	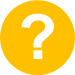	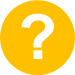	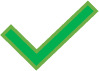	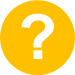	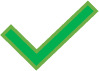	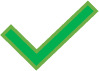	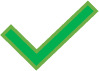
Poyurovsky et al. (2004)	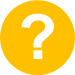	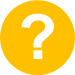	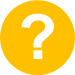	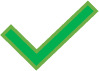	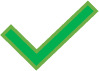	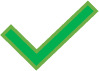	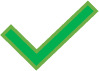	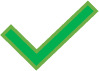
Poyurovsky et al. (2007)	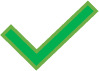	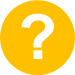	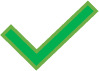	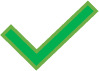	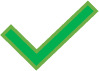	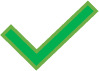	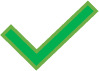	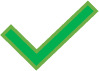
Poyurovsky et al. (2013)	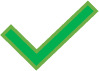	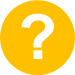	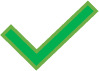	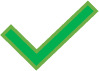	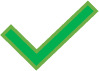	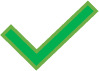	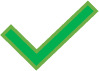	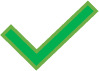
Wu et al. (2008)	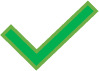	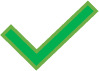	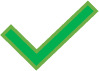	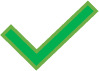	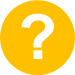	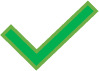	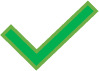	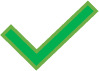

*Note:* 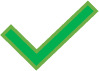 = Low risk of bias 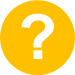 = Unclear risk of bias 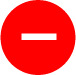 = High risk of bias.

### Prevention of weight gain

**Figure 2.**
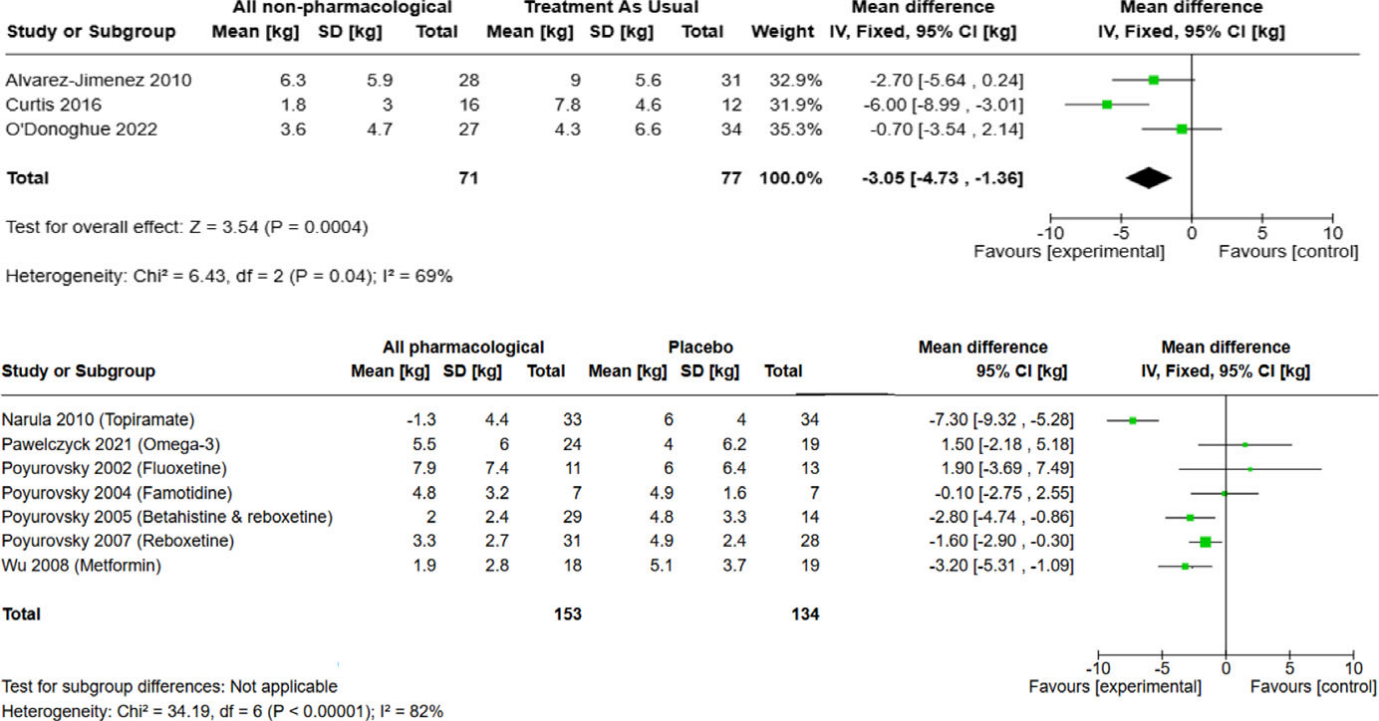
Forest plots of mean difference in weight gain at last of observation for interventions in adults.

Figure 2 presents forest plots of the last observed weight gain for each intervention in adults, excluding Kang et al.’s (2024) study due to the short follow-up of 5 days. Figure 2 also presents the results of our meta-analysis on the effects of behavioral interventions aimed at preventing AIWG.

When examined together using a fixed-effects model, non-pharmacological interventions for preventing AIWG resulted in a mean of 3.05 kg less weight gain than treatment-as-usual (95% CI, 1.36–4.73 kg). This result was robust to a sensitivity analysis of using a random-effects model (mean difference 3.06 kg, 95% CI, 0.11–6.01 kg), and to adjustment of the follow-up period to 3 months (the end of each intervention). A sensitivity analysis excluding Curtis et al.’s (2016) paper, due to its non-randomized design and high risk of bias, resulted in a non-significant impact of behavioral interventions on preventing AIWG, with a mean of 1.66 kg less weight gain than treatment-as-usual (95% CI −0.38 kg to 3.71 kg, p = 0.112). We judged the studies to be clinically homogeneous, with all three making use of a multidisciplinary team approach to preventing AIWG, and the I^2^ statistic was 71% (95% CI, 0%–99.27%, df = 2).

We considered the pharmacological interventions to be unsuitable for a meta-analysis, both due to clinical and statistical heterogeneity (I^2^ = 82%, 95% CI 56.7–96.3%, df = 6). Four studies showed evidence for an effect in reduction in weight gain: Metformin (mean difference of 3.2 kg, 95% CI 1.09–5.31 kg),[31] topiramate (mean difference of 7.2 kg, 95% CI 5.28–9.32 kg),[32] reboxetine (mean difference 1.6 kg, 95% CI 0.3–2.9 kg),[29] and a reboxetine/betahistine combination (mean difference 2.8 kg, 95% CI 0.86–4.74 kg) [30].

Kang et al.’s (2024) feasibility study of cTBS had a follow-up of only 5 days, however, they reported 1.14 kg (95% CI 0.3–1.98 kg) less weight gain in the intervention group, with a 5-day weight increase of 1.13 kg in the sham group.

### Metabolic markers

Most studies did not collect serum markers of metabolic health. Four studies presented data on serum lipids or triglycerides (Curtis et al., 2016; Kang et al., 2024; Narula et al., 2010; Pawełczyk et al., 2021), of which only the intervention with topiramate showed a favorable difference. The mean change in low-density lipoprotein (LDL) was significantly lower in the intervention group compared to placebo (mean difference 0.21 mM/L, 95% CI 0.07 mM/L to 0.35 mM/L) (Narula et al., 2010). Five studies presented data markers of changes in fasting glucose (Curtis et al., 2016; Kang et al., 2024; Narula et al., 2010; Pawełczyk et al., 2021; Wu et al., 2008). Of these, only the intervention of topiramate showed a significant reduction compared to control (mean difference 0.56 mM/L, 95% CI 0.41 mM/L to 0.72 mM/L) (Narula et al., 2010). Metformin showed a favorable response in fasting insulin and in the insulin-resistance index (Wu et al., 2008).

## Discussion

This is the first systematic review of interventions aiming to prevent antipsychotic-induced weight gain (AIWG), by intervening in drug-naïve or minimally exposed people. Our meta-analysis concords with reviews that advocate for a multidisciplinary team approach, with an emphasis on physical health, being a first-line treatment for people with FEP (Firth et al., 2019). Both fixed-effect and random-effect models showed that behavioral interventions were effective. Although excluding a study with a high risk of bias resulted in the meta-analysis showing no effect, this was largely driven by lower weight gain in the O’Donoghue et al. (2022) control group, who themselves received a multidisciplinary team intervention. Although such interventions would be biologically most effective if implemented immediately upon commencement of antipsychotic medication, patients must have recovered to an extent that they may engage in behavioral interventions, which may be difficult until after a period of antipsychotic exposure. Thus, in practice, these interventions should be commenced as soon as is practically possible. If a multidisciplinary team approach is not possible, pharmacological interventions show promise, but with low certainty of evidence. Pharmacological interventions as first-line may be reserved for high-risk individuals, who may be identified by a validated risk calculator (Carolan et al., 2025; Perry et al., 2021).

The statistical heterogeneity of behavioral interventions was below the 75% cut-off set out in our protocol, but the interventions differed clinically. Notably, the weight gain in the treatment-as-usual group was far lower in the O’Donoghue et al study compared to the others. O’Donoghue et al.’s (2022) control group received a full complement of multidisciplinary team input, similar to Curtis et al.’s (2016) KBIM intervention. Meanwhile, the control groups in Alvarez-Jimenez et al.’s (2006) and Curtis et al.’s (2016) studies received only standard medical reviews. This would appear to indicate that the service-wide MDT approach was itself effective in preventing weight gain. Although not specifically reported as confounders in these studies, both positive and negative symptoms may interfere with a patient’s ability to partake in behavioral interventions (Giordano et al., 2022; O’Keeffe, Conway, & McGuire, 2017).

Notably, only two studies of pharmacological interventions have been carried out since 2010, with one of these being a secondary analysis of a prior study (Pawełczyk et al., 2016). The limited number of identified studies is most likely due to the difficulty in recruiting a drug-naïve FEP cohort for interventional studies. Patients must have suffered a recent episode of severe mental illness but have stabilized sufficiently within the 4-week window to provide informed consent for study participation. In routine clinical practice, the number of individuals eligible for such intervention substantially surpasses those who can be feasibly enrolled in RCTs.

Only four studies assessing the impact of interventions on lipid profile or glucose regulation were identified, with most reporting these as secondary outcomes with limited statistical power. Fasting glucose, which is not consistently altered in early insulin resistance, was the primary measure of glucose homeostasis in nearly all intervention studies, and its relevance to glucose dysregulation in FEP patients remains unclear (Perry et al., 2016; Pillinger, D’ambrosio, McCutcheon, & Howes, 2019). Notably, only one study assessed for insulin resistance (Wu et al., 2008), a factor which has consistently been shown to be elevated at FEP (Perry et al., 2016; Pillinger et al., 2019).

Pharmacological interventions were notable for both statistical heterogeneity and heterogeneity in the medications used, with the latter likely explaining the former. Other possible explanations for this statistical heterogeneity include variations in study duration and participant ethnicity (Pillinger et al., 2020). The pharmacological interventions identified made use of a variety of mechanisms to prevent AIWG. Metformin likely ameliorates weight gain by improving insulin sensitivity, reducing hepatic glucose production, and lowering appetite (LaMoia & Shulman, 2021). Topiramate is thought to reduce weight gain primarily through its effect on appetite and by increasing energy expenditure (Verrotti et al., 2011). The different monoamine modulators used by Poyurovsky et al. are less commonly recognized as methods to prevent AIWG, but the apparent effectiveness of reboxetine is intriguing, given the high rates of antidepressant prescriptions in FEP services (Hamina et al., 2023; Robinson et al., 2015).

While our review has not added clear evidence regarding the FEP population specifically, pharmacological treatment has been shown to be effective in treating metabolic dysfunction in patients with psychotic disorders (Ye et al., 2023). Metformin has shown particular promise (Mansuri et al., 2022; Peng et al., 2025), and its use has been recommended as part of routine care (Carolan et al., 2025). Indeed, a meta-analysis of studies that commenced metformin within 1 year of antipsychotic initiation showed its efficacy (Yu et al., 2024). However, pharmacological interventions carry short and long-term side-effects, which range from mild to severe. The most bothersome side effect of metformin is gastrointestinal upset, but its use can also lead to pancreatitis, lactic acidosis, and hepatotoxicity (Shurrab & Arafa, 2020). Similarly, topiramate has shown a positive impact on both symptomatology and weight in schizophrenia, but has led to notable cognitive side effects. These cognitive effects have contributed to a decline in topiramate use as a first-line treatment for epilepsy (Mula & Trimble, 2009; Okuyama et al., 2016). There has been limited research into the long-term outcomes of such pharmacological interventions, meaning that their prescription and these side-effects may last indefinitely.

Behavioral interventions have similarly shown promise in improving metabolic health in individuals with psychotic disorders (Naslund et al., 2017). They offer several advantages that are not typically associated with pharmacological approaches, such as fostering skills that would extend beyond the time-period of a medication prescription. Behavioral interventions are also more likely to empower patients and engender a sense of autonomy, self-actualization, and self-esteem, which are crucial in overcoming the stigma associated with psychotic disorders (Firth et al., 2019).

Negative symptoms, periods of mental illness, chronic stress, and side effects of antipsychotic medication may all contribute to difficulties in initiating and adhering to behavioral treatments (Firth et al., 2018). However, amotivation and apathy do not preclude an individual with serious mental illness from engaging in and benefiting from exercise (Carpiniello et al., 2013; Firth et al., 2016). Indeed, exercise appears to be effective in alleviating these same negative symptoms (Kim, Lee, & Kang, 2023). Long-term adherence to such interventions has been demonstrated to wane as the structure and support of the intervention are withdrawn. However, this effect may be greater in patients with chronic schizophrenia rather than FEP, which offers a critical window to implement interventions (Carpiniello et al., 2013; Deighton & Addington, 2014). An interesting study not included in our review, as we did not consider it a true intervention, showed reduced weight gain in patients taking orally disintegrating olanzapine rather than a standard olanzapine tablet (Arranz et al., 2007). Various mechanisms have been proposed, including differences in absorption, activation of serotonergic receptors in the pylorus, and effects on taste receptors and gastrointestinal hormones (Karagianis et al., 2008). Of note, this difference in weight gain was not replicated in a larger trial of olanzapine-naïve, though not antipsychotic-naïve, individuals with schizophrenia (Kusumi et al., 2012). The impact of this strategy on drug-naïve individuals experiencing a FEP may be worth exploring.

Although not included in our review, Glucagon-Like Peptide 1 (GLP-1) agonists will likely play a large role in the future of AIWG treatment (Ganeshalingam et al., 2024), given their remarkable results in inducing weight loss in obese and diabetic patients (Bak et al., 2024). The pilot study of continuous theta burst stimulation by Kang et al. showed tolerability and a significant reduction in weight gain, but was limited by a very short follow-up period of 5 days (Kang et al., 2024). Their hypothesis, that this effect was mediated by improved cognitive restraint on food, is intriguing and offers a promising avenue for future research.

## Conclusion

Despite the importance of preventing weight gain in FEP, there have been few studies investigating this topic. Additionally, many are limited by small sample sizes and short study durations. Despite these limitations, several interventions appear promising in addressing a clinically significant contributor to excess morbidity and mortality in this population. Our results indicate that multidisciplinary team interventions appear to be effective in preventing weight gain in FEP and, given their lack of adverse effects, should ideally be offered to all patients. Future research should investigate true prevention of harm to metabolic health by antipsychotics, through recruitment of drug-naïve or minimally exposed patients, and by performing comprehensive assessments of metabolic health.

## Supporting information

10.1017/S0033291726104139.sm001 O’Mahony et al. supplementary material 1 O’mahony et al. supplementary material

10.1017/S0033291726104139.sm002 O’Mahony et al. supplementary material 2 O’mahony et al. supplementary material

## Supplementary material

The supplementary material for this article can be found at http://doi.org/10.1017/S0033291726104139.
