# Supplementary material for: Interventions to prevent antipsychotic-induced weight gain and metabolic complications in individuals with a first-episode psychosis and minimal antipsychotic exposure: a systematic review and meta-analysis
Source: Psychol Med. 2026 Apr 10;56:e101. doi: 10.1017/S0033291726104139 (PMC13079223; doi:10.1017/S0033291726104139)
Supplement: O’Mahony et al. supplementary material 1 — O’mahony et al. supplementary material [file S0033291726104139sup001.docx]

**Search Strategy 1: Ovid Platform (MEDLINE, EMBASE, PsycInfo)**

The following strategy was run in each database on the Ovid platform. Syntax variations between databases (e.g., MeSH for MEDLINE, Emtree for EMBASE) were adapted automatically by the platform where possible.

| **#** | **Search Terms** |
| --- | --- |
| 1 | schizophrenia.mp. or exp schizophrenia spectrum disorder/ or exp schizophrenia/ |
| 2 | psychosis.mp. or exp psychosis/ |
| 3 | psychotic.mp. |
| **4** | **1 or 2 or 3** |
| 5 | weight.mp. or exp weight/ |
| 6 | bmi.mp. or exp body mass/ or obesity/ |
| 7 | exp waist hip ratio/ or waist.mp. or exp waist circumference/ |
| 8 | hypertension.mp. or exp systolic hypertension/ or exp hypertension/ |
| 9 | (hyperlipidemia.mp. or exp hyperlipidemia/) or (hyperlipidaemia.mp. or exp hyperlipidaemia/) |
| 10 | cholesterol.mp. or exp cholesterol/ |
| 11 | exp insulin/ or exp glucose/ or exp diabetes mellitus/ or glucose.mp. or exp glucose blood level/ |
| 12 | metabolic syndrome.mp. or exp metabolic syndrome X/ |
| **13** | **5 or 6 or 7 or 8 or 9 or 10 or 11 or 12** |
| 14 | first episode.mp. |
| 15 | early.mp. |
| **16** | **14 or 15** |
| **17** | **4 and 13 and 16** |
| 18 | limit 17 to (clinical trial or randomized controlled trial or controlled clinical trial or multicenter study or pilot study or phase 1 clinical trial or phase 2 clinical trial or phase 3 clinical trial or phase 4 clinical trial or systematic review or meta analysis) |

**Syntax Key:**

- .mp. = multi-purpose search (searches title, abstract, subject headings, and other fields)
- exp ... / = 'explodes' the subject heading to include all more specific terms in the hierarchy
- / = subject heading

**Search Strategy 2: EBSCOhost Platform (CINAHL)**

The following strategy was run in CINAHL on the EBSCOhost platform.

| **#** | **Search Terms** |
| --- | --- |
| S1 | (MH "Schizophrenia Spectrum and Other Psychotic Disorders+") OR (MH "Schizophrenia+") OR TI (schizophrenia or psychosis or psychotic) OR AB (schizophrenia or psychosis or psychotic) |
| S2 | (MH "Body Weight+") OR (TI weight OR AB weight) OR (MH "Body Mass Index+") OR (MH "Obesity+") OR (TI bmi OR AB bmi) OR (MH "Waist-Hip Ratio+") OR (MH "Waist Circumference+") OR (TI waist OR AB waist) OR (MH "Hypertension+") OR (TI hypertension OR AB hypertension) OR (MH "Hyperlipidemia+") OR (TI (hyperlipidemia or hyperlipidaemia) OR AB (hyperlipidemia or hyperlipidaemia)) OR (MH "Cholesterol+") OR (TI cholesterol OR AB cholesterol) OR (MH "Insulin+") OR (MH "Glucose+") OR (MH "Diabetes Mellitus+") OR (MH "Blood Glucose+") OR (TI glucose OR AB glucose) OR (MH "Metabolic Syndrome X+") OR (TI "metabolic syndrome" OR AB "metabolic syndrome") |
| S3 | TI ("first episode" or early) OR AB ("first episode" or early) |
| **S4** | **S1 AND S2 AND S3** |
| S5 | S4 AND (PT "Clinical Trial" OR PT "Randomized Controlled Trial" OR PT "Pilot Study" OR PT "Systematic Review" OR PT "Meta Analysis") |

**Syntax Key:**

- MH "..." = CINAHL Subject Heading
- + = 'explodes' the subject heading
- TI (...) = search in Title
- AB (...) = search in Abstract
- PT "..." = Publication Type
